# Aqua­(2,2′-diamino-4,4′-bi-1,3-thia­zole-κ^2^ *N* ^3^,*N* ^3′^)(pyridine-2,6-dicarboxyl­ato-κ^3^ *O* ^2^,*N*,*O* ^6^)zinc tetra­hydrate

**DOI:** 10.1107/S1600536811015145

**Published:** 2011-05-07

**Authors:** Yan-Li Wang, Guang-Jun Chang, Bing-Xin Liu

**Affiliations:** a Department of Chemistry, Shanghai University, People’s Republic of China

## Abstract

The title compound, [Zn(C_7_H_3_NO_4_)(C_6_H_6_N_4_S_2_)(H_2_O)]·4H_2_O, assumes a distorted octa­hedral coordination geometry around the Zn^2+^ cation, formed by a diamino­bithia­zole (DABT) mol­ecule, a pyridine-2,6-dicarboxyl­ate anion and a water mol­ecule. The pyridine-2,6-dicarboxyl­ate anion chelates to the Zn^II^ atom with a facial configuration. Within the chelating DABT ligand, the two thia­zole rings are twisted by a dihedral angle of 14.52 (8)° with respect to each other. O—H⋯O and N—H⋯O hydrogen bonds occur in the crystal structure.

## Related literature

For potential applications of transition metal complexes of 2,2′-diamino-4,4′-bi-1,3-thia­zole (DABT), see: Sun *et al.* (1997 ▶). For general background to metal complexes with DABT, see: Liu *et al.* (2003 ▶). For related structures, see: Liu & Xu (2004 ▶, 2005 ▶); Liu *et al.* (2005 ▶).
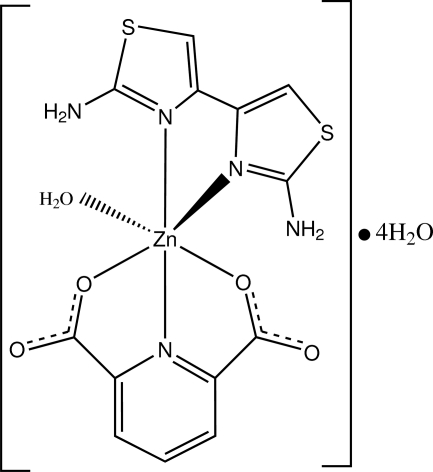


## Experimental

### 

#### Crystal data

[Zn(C_7_H_3_NO_4_)(C_6_H_6_N_4_S_2_)(H_2_O)]·4H_2_O
*M* *_r_* = 518.82
Monoclinic, 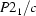

*a* = 10.0529 (19) Å
*b* = 7.0833 (13) Å
*c* = 27.720 (6) Å
β = 93.960 (3)°
*V* = 1969.2 (7) Å^3^
*Z* = 4
Mo *K*α radiation
μ = 1.52 mm^−1^
*T* = 295 K
0.25 × 0.20 × 0.15 mm

#### Data collection

Bruker SMART APEX diffractometer
Absorption correction: multi-scan (*SADABS*; Sheldrick, 1996 ▶) *T*_min_ = 0.701, *T*_max_ = 0.796
9851 measured reflections
3471 independent reflections
2238 reflections with *I* > 2σ(*I*)
*R*_int_ = 0.074

#### Refinement

*R*[*F* ^2^ > 2σ(*F* ^2^)] = 0.052
*wR*(*F* ^2^) = 0.119
*S* = 1.03
3471 reflections
281 parameters
H-atom parameters constrained
Δρ_max_ = 0.40 e Å^−3^
Δρ_min_ = −0.60 e Å^−3^

### 

Data collection: *SMART* (Bruker, 2004 ▶); cell refinement: *SAINT* (Bruker, 2004 ▶); data reduction: *SAINT*; program(s) used to solve structure: *SIR92* (Altomare *et al.*, 1993 ▶); program(s) used to refine structure: *SHELXL97* (Sheldrick, 2008 ▶); molecular graphics: *ORTEP-3 for Windows* (Farrugia, 1997 ▶); software used to prepare material for publication: *WinGX* (Farrugia, 1999 ▶).

## Supplementary Material

Crystal structure: contains datablocks I, global. DOI: 10.1107/S1600536811015145/ff2006sup1.cif

Structure factors: contains datablocks I. DOI: 10.1107/S1600536811015145/ff2006Isup2.hkl

Additional supplementary materials:  crystallographic information; 3D view; checkCIF report

## Figures and Tables

**Table 1:** Selected bond lengths (Å)

Zn—N21	2.064 (4)
Zn—N11	2.092 (4)
Zn—N13	2.129 (4)
Zn—O1	2.213 (3)
Zn—O23	2.232 (4)
Zn—O21	2.260 (4)

**Table 2:** Hydrogen-bond geometry (Å, °)

*D*—H⋯*A*	*D*—H	H⋯*A*	*D*⋯*A*	*D*—H⋯*A*
O1—H1*A*⋯O22^i^	0.97	1.84	2.778 (5)	163
O1—H1*B*⋯O1*W*	0.96	1.87	2.827 (6)	174
O1*W*—H1*WA*⋯O4*W*^ii^	0.91	2.10	2.812 (6)	134
O1*W*—H1*WB*⋯O2*W*^i^	0.80	2.10	2.775 (6)	142
O2*W*—H2*WA*⋯O22	0.82	1.93	2.692 (6)	155
O2*W*—H2*WB*⋯O4*W*^iii^	0.86	1.97	2.830 (6)	178
O3*W*—H3*WA*⋯O24	0.94	1.94	2.880 (6)	174
O3*W*—H3*WB*⋯O24^iv^	0.96	1.80	2.694 (6)	153
O4*W*—H4*WA*⋯O2*W*^ii^	0.91	2.02	2.863 (6)	153
O4*W*—H4*WB*⋯O3*W*	0.88	1.92	2.783 (6)	167
N12—H12*A*⋯O1	0.97	2.00	2.873 (6)	149
N12—H12*B*⋯O21^v^	0.83	2.19	2.984 (5)	161
N14—H14*A*⋯O3*W*^vi^	0.88	2.44	3.043 (6)	126
N14—H14*B*⋯O1*W*^vii^	0.86	2.19	3.022 (6)	162

Symmetry codes: (i) 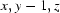; (ii) 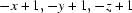; (iii) 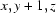; (iv) 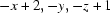; (v) 
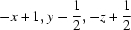
; (vi) 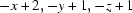; (vii) 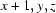.

**Fig. 1.**
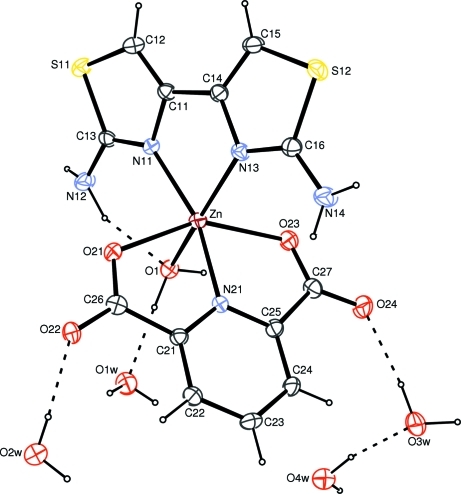
The molecular structure of (I) with 30% probability displacement ellipsoids (arbitrary spheres for H atoms), dashed lines showing the hydrogen bonding within the complex.

**Fig. 2.**
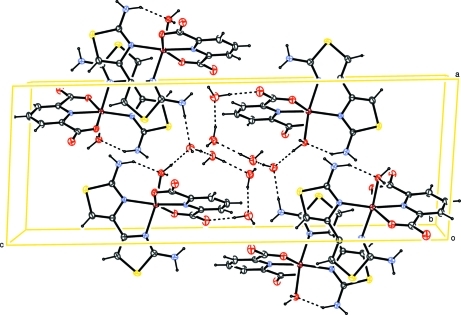
The hydrogen bonding diagram with 30% probability displacement ellipsoids (arbitrary spheres for H atoms), dashed lines indicate the hydrogen bonding.

## supplementary crystallographic information

### Comment

Transition metal complexes of 2,2'-diamino-4,4'-bi-1,3-thiazole (DABT) have shown potential application in the field of soft magnetic material (Sun *et al.*, 1997). As part of serial structural investigation of metal complexes with DABT (Liu *et al.*, 2003), the title Zn^II^ complex was recently prepared and its X-ray structure is presented here.

The molecular structure of the title compound is shown in Fig. 1. The complex has a distorted octahedral coordinatation geometry formed by a DABT ligand, a pyridine-2,6-dicarboxylate anion and a coordinated water molecule.

Thiazole rings of DABT are not coplanar as same as in other complexes we have reported, the dihedral angle between the two thiazole rings is 14.51 (8) °. It is similar to the 17.23 (7) ° found in [Cr(C_4_H_5_NO_4_)(C_6_H_6_N_4_S_2_)(H_2_O)]Cl^.^H_2_O, (Liu & Xu, 2004). The distances of C16—N14 [1.335 (4) Å] and C16—N13[1.324 (4) Å] imply the existence of electron delocalization between thiazole rings and amino groups. This feature of electron delocalization of DABT can be found in some DABT complexes of Mn(II) (Liu & Xu, 2005), Co(II) (Liu *et al.*, 2005), we have reported. The tridentate pyridine-2,6-dicarboxylate anion chelates to the Zn^II^ atom with a facial configuration with the maximum atomic deviation of 0.082 (3) Å (N21) to the main plane defined by C21 C22 C23 C24 C25 C26 C27 N21 O21 O22 O23 O24.

The extensive hydrogen bonding between lattice water molecules, complex and lattice water helps to stabilize the crystal structure as shown in Fig. 2. and Table 1.

### Experimental

An aqueous solution (20 ml) containing DABT (1 mmol) and ZnCl_2_ (1 mmol) was mixed with an aqueous solution (10 ml) of pyridine-2,6-dicarboxylic acid (1 mmol) and NaOH (2 mmol). The mixture was refluxed for 5 h. After cooling to room temperature the solution was filtered. Single crystals of (I) were obtained from the filtrate after 10 d.

### Refinement

H atoms on carbon atoms were placed in calculated positions, with C—H distances = 0.93 Å (aromatic), and were included in the final cycles of refinement in riding mode with *U*_iso_(H) = 1.2*U*_eq_ of the carrier atoms. H atoms of amino group of DABT, coordinated water and lattice water were located in a difference Fourier map and included in the structure factor calculations with fixed positional and isotropic displacement parameters *U*_iso_(H) = 1.2*U*_eq_(N) and 1.5*U*_eq_(O) of the carrier atoms.

### Crystal data

**Table:**

[Zn(C_7_H_3_NO_4_)(C_6_H_6_N_4_S_2_)(H_2_O)]·4H_2_O	*F*(000) = 1064
*M**_r_* = 518.82	*D*_x_ = 1.750 Mg m^−^^3^
Monoclinic, *P*2_1_/*c*	Mo *K*α radiation, λ = 0.71073 Å
Hall symbol: -P 2ybc	Cell parameters from 3380 reflections
*a* = 10.0529 (19) Å	θ = 2.0–25.0°
*b* = 7.0833 (13) Å	µ = 1.52 mm^−^^1^
*c* = 27.720 (6) Å	*T* = 295 K
β = 93.960 (3)°	Prism, yellow
*V* = 1969.2 (7) Å^3^	0.25 × 0.20 × 0.15 mm
*Z* = 4	

### Data collection

**Table:**

Bruker SMART APEX diffractometer	3471 independent reflections
Radiation source: fine-focus sealed tube	2238 reflections with *I* > 2σ(*I*)
graphite	*R*_int_ = 0.074
Detector resolution: 10.0 pixels mm^-1^	θ_max_ = 25.0°, θ_min_ = 2.4°
ω scans	*h* = −11→11
Absorption correction: multi-scan (*SADABS*; Sheldrick, 1996)	*k* = −8→8
*T*_min_ = 0.701, *T*_max_ = 0.796	*l* = −22→32
9851 measured reflections	

### Refinement

**Table:**

Refinement on *F*^2^	Primary atom site location: structure-invariant direct methods
Least-squares matrix: full	Secondary atom site location: difference Fourier map
*R*[*F*^2^ > 2σ(*F*^2^)] = 0.052	Hydrogen site location: inferred from neighbouring sites
*wR*(*F*^2^) = 0.119	H-atom parameters constrained
*S* = 1.03	*w* = 1/[σ^2^(*F*_o_^2^) + (0.0374*P*)^2^ + 1.648*P*] where *P* = (*F*_o_^2^ + 2*F*_c_^2^)/3
3471 reflections	(Δ/σ)_max_ < 0.001
281 parameters	Δρ_max_ = 0.40 e Å^−^^3^
0 restraints	Δρ_min_ = −0.60 e Å^−^^3^

### Special details

**Table:**

Geometry. All e.s.d.'s (except the e.s.d. in the dihedral angle between two l.s. planes) are estimated using the full covariance matrix. The cell e.s.d.'s are taken into account individually in the estimation of e.s.d.'s in distances, angles and torsion angles; correlations between e.s.d.'s in cell parameters are only used when they are defined by crystal symmetry. An approximate (isotropic) treatment of cell e.s.d.'s is used for estimating e.s.d.'s involving l.s. planes.
Refinement. Refinement of *F*^2^ against ALL reflections. The weighted *R*-factor *wR* and goodness of fit *S* are based on *F*^2^, conventional *R*-factors *R* are based on *F*, with *F* set to zero for negative *F*^2^. The threshold expression of *F*^2^ > σ(*F*^2^) is used only for calculating *R*-factors(gt) *etc*. and is not relevant to the choice of reflections for refinement. *R*-factors based on *F*^2^ are statistically about twice as large as those based on *F*, and *R*- factors based on ALL data will be even larger.

### Fractional atomic coordinates and isotropic or equivalent isotropic displacement parameters (Å^2^)

**Table:**

	*x*	*y*	*z*	*U*_iso_*/*U*_eq_	
Zn	0.79367 (6)	0.53096 (9)	0.32687 (2)	0.0278 (2)	
O1	0.6001 (3)	0.3881 (5)	0.33361 (13)	0.0323 (9)	
H1A	0.6206	0.2602	0.3443	0.031 (15)*	
H1B	0.5456	0.4465	0.3567	0.06 (2)*	
O21	0.6938 (3)	0.8166 (5)	0.32814 (13)	0.0322 (9)	
O22	0.6230 (4)	1.0365 (5)	0.37777 (13)	0.0352 (9)	
O23	0.8776 (4)	0.2804 (5)	0.36725 (13)	0.0330 (9)	
O24	0.9366 (4)	0.1804 (6)	0.44238 (14)	0.0460 (11)	
N11	0.7611 (4)	0.5270 (6)	0.25153 (14)	0.0249 (10)	
N12	0.5302 (4)	0.4822 (7)	0.23428 (16)	0.0401 (13)	
H12A	0.5190	0.4432	0.2673	0.048*	
H12B	0.4789	0.4457	0.2116	0.048*	
N13	0.9917 (4)	0.5795 (6)	0.30709 (15)	0.0270 (11)	
N14	1.1350 (4)	0.5784 (7)	0.37784 (17)	0.0434 (13)	
H14A	1.0773	0.6054	0.3991	0.052*	
H14B	1.2189	0.5943	0.3857	0.052*	
N21	0.7907 (4)	0.6039 (6)	0.39887 (15)	0.0238 (10)	
S11	0.68935 (14)	0.5508 (2)	0.16108 (5)	0.0368 (4)	
S12	1.23955 (13)	0.5564 (2)	0.29119 (5)	0.0348 (4)	
C11	0.8759 (5)	0.5611 (7)	0.22697 (18)	0.0250 (12)	
C12	0.8558 (5)	0.5786 (8)	0.1793 (2)	0.0333 (14)	
H12	0.9225	0.6026	0.1585	0.040*	
C13	0.6547 (5)	0.5153 (7)	0.22092 (18)	0.0265 (12)	
C14	1.0006 (5)	0.5681 (7)	0.25667 (19)	0.0255 (12)	
C15	1.1253 (5)	0.5557 (8)	0.2422 (2)	0.0323 (13)	
H15	1.1467	0.5478	0.2102	0.039*	
C16	1.1095 (5)	0.5723 (8)	0.3291 (2)	0.0306 (13)	
C21	0.7370 (5)	0.7687 (7)	0.41205 (18)	0.0243 (12)	
C22	0.7300 (6)	0.8163 (8)	0.45964 (19)	0.0348 (14)	
H22	0.6938	0.9313	0.4682	0.042*	
C23	0.7783 (6)	0.6894 (8)	0.4950 (2)	0.0364 (14)	
H23	0.7755	0.7193	0.5276	0.044*	
C24	0.8303 (5)	0.5195 (7)	0.4814 (2)	0.0324 (14)	
H24	0.8614	0.4324	0.5046	0.039*	
C25	0.8355 (5)	0.4801 (7)	0.43272 (19)	0.0270 (12)	
C26	0.6810 (5)	0.8848 (8)	0.3697 (2)	0.0287 (13)	
C27	0.8889 (5)	0.2973 (7)	0.4127 (2)	0.0298 (13)	
O1W	0.4314 (4)	0.5366 (7)	0.40173 (16)	0.0453 (11)	
H1WA	0.4454	0.6614	0.4085	0.06 (2)*	
H1WB	0.4538	0.4785	0.4256	0.08 (3)*	
O2W	0.5018 (4)	1.2043 (6)	0.45001 (16)	0.0533 (12)	
H2WA	0.5563	1.1778	0.4303	0.06 (2)*	
H2WB	0.5413	1.1846	0.4782	0.05 (2)*	
O3W	0.9102 (4)	0.1298 (5)	0.54438 (17)	0.0485 (12)	
H3WA	0.9229	0.1380	0.5111	0.11 (3)*	
H3WB	0.9730	0.0390	0.5580	0.051 (18)*	
O4W	0.6334 (4)	0.1492 (6)	0.54252 (14)	0.0465 (11)	
H4WA	0.6074	0.0351	0.5539	0.14 (4)*	
H4WB	0.7192	0.1338	0.5388	0.05 (2)*	

### Atomic displacement parameters (Å^2^)

**Table:**

	*U*^11^	*U*^22^	*U*^33^	*U*^12^	*U*^13^	*U*^23^
Zn	0.0276 (4)	0.0333 (4)	0.0226 (4)	0.0023 (3)	0.0028 (3)	0.0000 (3)
O1	0.034 (2)	0.032 (2)	0.031 (2)	0.0021 (18)	0.0031 (18)	0.0038 (18)
O21	0.034 (2)	0.040 (2)	0.022 (2)	0.0082 (18)	−0.0013 (17)	0.0010 (18)
O22	0.045 (2)	0.024 (2)	0.037 (2)	0.0128 (19)	0.0054 (19)	0.0033 (18)
O23	0.037 (2)	0.028 (2)	0.035 (2)	0.0057 (17)	0.0031 (19)	−0.0012 (18)
O24	0.065 (3)	0.033 (2)	0.040 (3)	0.022 (2)	0.004 (2)	0.005 (2)
N11	0.025 (2)	0.027 (3)	0.022 (2)	0.001 (2)	0.0027 (19)	0.003 (2)
N12	0.030 (3)	0.065 (4)	0.024 (3)	0.000 (3)	−0.003 (2)	0.001 (2)
N13	0.022 (2)	0.031 (3)	0.028 (3)	−0.0009 (19)	0.003 (2)	0.000 (2)
N14	0.023 (3)	0.073 (4)	0.035 (3)	−0.003 (2)	0.004 (2)	−0.002 (3)
N21	0.021 (2)	0.026 (3)	0.024 (3)	0.0024 (19)	0.0020 (19)	0.000 (2)
S11	0.0403 (9)	0.0476 (10)	0.0221 (8)	0.0015 (7)	−0.0013 (6)	0.0025 (7)
S12	0.0238 (7)	0.0401 (9)	0.0412 (9)	0.0012 (6)	0.0064 (7)	0.0010 (7)
C11	0.026 (3)	0.025 (3)	0.024 (3)	0.000 (2)	0.002 (2)	−0.005 (2)
C12	0.036 (3)	0.038 (4)	0.027 (3)	−0.002 (3)	0.008 (3)	0.002 (3)
C13	0.028 (3)	0.029 (3)	0.023 (3)	0.006 (2)	0.002 (2)	−0.007 (2)
C14	0.031 (3)	0.021 (3)	0.025 (3)	0.003 (2)	0.004 (2)	−0.002 (2)
C15	0.036 (3)	0.035 (3)	0.027 (3)	0.000 (3)	0.011 (3)	0.003 (3)
C16	0.030 (3)	0.033 (3)	0.030 (3)	−0.001 (3)	0.004 (3)	0.001 (3)
C21	0.027 (3)	0.024 (3)	0.022 (3)	0.000 (2)	0.002 (2)	−0.001 (2)
C22	0.044 (4)	0.031 (3)	0.029 (3)	0.013 (3)	0.004 (3)	−0.003 (3)
C23	0.046 (4)	0.043 (4)	0.020 (3)	0.006 (3)	0.007 (3)	−0.001 (3)
C24	0.044 (3)	0.026 (3)	0.027 (3)	0.011 (3)	0.003 (3)	0.006 (3)
C25	0.027 (3)	0.025 (3)	0.029 (3)	0.000 (2)	0.004 (2)	0.006 (3)
C26	0.027 (3)	0.027 (3)	0.032 (4)	−0.006 (3)	0.003 (3)	−0.003 (3)
C27	0.034 (3)	0.019 (3)	0.038 (4)	0.001 (2)	0.006 (3)	0.002 (3)
O1W	0.045 (3)	0.046 (3)	0.044 (3)	0.007 (2)	0.001 (2)	−0.001 (2)
O2W	0.059 (3)	0.067 (3)	0.035 (3)	0.023 (2)	0.010 (3)	0.010 (2)
O3W	0.054 (3)	0.040 (3)	0.052 (3)	0.021 (2)	0.008 (2)	0.006 (2)
O4W	0.050 (3)	0.050 (3)	0.040 (3)	0.004 (2)	0.006 (2)	−0.002 (2)

### Geometric parameters (Å, °)

**Table:**

Zn—N21	2.064 (4)	S11—C13	1.737 (5)
Zn—N11	2.092 (4)	S12—C15	1.717 (6)
Zn—N13	2.129 (4)	S12—C16	1.736 (5)
Zn—O1	2.213 (3)	C11—C12	1.328 (7)
Zn—O23	2.232 (4)	C11—C14	1.452 (7)
Zn—O21	2.260 (4)	C12—H12	0.9300
O1—H1A	0.9713	C14—C15	1.346 (7)
O1—H1B	0.9633	C15—H15	0.9300
O21—C26	1.264 (6)	C21—C22	1.368 (7)
O22—C26	1.250 (6)	C21—C26	1.510 (7)
O23—C27	1.264 (6)	C22—C23	1.393 (7)
O24—C27	1.239 (6)	C22—H22	0.9300
N11—C13	1.321 (6)	C23—C24	1.375 (7)
N11—C11	1.401 (6)	C23—H23	0.9300
N12—C13	1.351 (6)	C24—C25	1.382 (7)
N12—H12A	0.9708	C24—H24	0.9300
N12—H12B	0.8256	C25—C27	1.521 (7)
N13—C16	1.294 (7)	O1W—H1WA	0.9122
N13—C14	1.409 (6)	O1W—H1WB	0.7978
N14—C16	1.359 (7)	O2W—H2WA	0.8216
N14—H14A	0.8749	O2W—H2WB	0.8624
N14—H14B	0.8645	O3W—H3WA	0.9418
N21—C25	1.339 (6)	O3W—H3WB	0.9604
N21—C21	1.347 (6)	O4W—H4WA	0.9116
S11—C12	1.725 (6)	O4W—H4WB	0.8825
			
N21—Zn—N11	163.19 (16)	C11—C12—S11	111.1 (4)
N21—Zn—N13	106.55 (16)	C11—C12—H12	124.5
N11—Zn—N13	80.18 (16)	S11—C12—H12	124.5
N21—Zn—O1	87.78 (14)	N11—C13—N12	124.0 (5)
N11—Zn—O1	90.03 (15)	N11—C13—S11	113.4 (4)
N13—Zn—O1	159.90 (15)	N12—C13—S11	122.5 (4)
N21—Zn—O23	75.18 (15)	C15—C14—N13	115.0 (5)
N11—Zn—O23	121.17 (15)	C15—C14—C11	127.9 (5)
N13—Zn—O23	85.97 (15)	N13—C14—C11	117.0 (4)
O1—Zn—O23	84.17 (13)	C14—C15—S12	110.5 (4)
N21—Zn—O21	74.03 (14)	C14—C15—H15	124.7
N11—Zn—O21	89.34 (14)	S12—C15—H15	124.7
N13—Zn—O21	106.47 (15)	N13—C16—N14	124.8 (5)
O1—Zn—O21	90.79 (14)	N13—C16—S12	114.8 (4)
O23—Zn—O21	148.96 (13)	N14—C16—S12	120.4 (4)
Zn—O1—H1A	106.4	N21—C21—C22	121.6 (5)
Zn—O1—H1B	113.8	N21—C21—C26	113.3 (4)
H1A—O1—H1B	108.5	C22—C21—C26	125.0 (5)
C26—O21—Zn	115.4 (3)	C21—C22—C23	118.7 (5)
C27—O23—Zn	115.5 (3)	C21—C22—H22	120.6
C13—N11—C11	110.9 (4)	C23—C22—H22	120.6
C13—N11—Zn	134.9 (3)	C24—C23—C22	119.5 (5)
C11—N11—Zn	113.9 (3)	C24—C23—H23	120.3
C13—N12—H12A	118.5	C22—C23—H23	120.3
C13—N12—H12B	112.8	C23—C24—C25	119.1 (5)
H12A—N12—H12B	121.5	C23—C24—H24	120.5
C16—N13—C14	110.3 (4)	C25—C24—H24	120.5
C16—N13—Zn	135.5 (4)	N21—C25—C24	121.2 (5)
C14—N13—Zn	111.7 (3)	N21—C25—C27	114.3 (5)
C16—N14—H14A	126.0	C24—C25—C27	124.5 (5)
C16—N14—H14B	111.6	O22—C26—O21	124.7 (5)
H14A—N14—H14B	118.9	O22—C26—C21	118.9 (5)
C25—N21—C21	119.9 (4)	O21—C26—C21	116.4 (5)
C25—N21—Zn	119.2 (3)	O24—C27—O23	127.1 (5)
C21—N21—Zn	120.8 (3)	O24—C27—C25	117.2 (5)
C12—S11—C13	89.5 (3)	O23—C27—C25	115.7 (5)
C15—S12—C16	89.3 (3)	H1WA—O1W—H1WB	107.4
C12—C11—N11	115.2 (5)	H2WA—O2W—H2WB	106.1
C12—C11—C14	128.9 (5)	H3WA—O3W—H3WB	107.3
N11—C11—C14	116.0 (4)	H4WA—O4W—H4WB	103.6

### Hydrogen-bond geometry (Å, °)

**Table:**

*D*—H···*A*	*D*—H	H···*A*	*D*···*A*	*D*—H···*A*
O1—H1A···O22^i^	0.97	1.84	2.778 (5)	163
O1—H1B···O1W	0.96	1.87	2.827 (6)	174
O1W—H1WA···O4W^ii^	0.91	2.10	2.812 (6)	134
O1W—H1WB···O2W^i^	0.80	2.10	2.775 (6)	142
O2W—H2WA···O22	0.82	1.93	2.692 (6)	155
O2W—H2WB···O4W^iii^	0.86	1.97	2.830 (6)	178
O3W—H3WA···O24	0.94	1.94	2.880 (6)	174
O3W—H3WB···O24^iv^	0.96	1.80	2.694 (6)	153
O4W—H4WA···O2W^ii^	0.91	2.02	2.863 (6)	153
O4W—H4WB···O3W	0.88	1.92	2.783 (6)	167
N12—H12A···O1	0.97	2.00	2.873 (6)	149
N12—H12B···O21^v^	0.83	2.19	2.984 (5)	161
N14—H14A···O3W^vi^	0.88	2.44	3.043 (6)	126
N14—H14B···O1W^vii^	0.86	2.19	3.022 (6)	162

Symmetry codes: (i) *x*, *y*−1, *z*; (ii) −*x*+1, −*y*+1, −*z*+1; (iii) *x*, *y*+1, *z*; (iv) −*x*+2, −*y*, −*z*+1; (v) −*x*+1, *y*−1/2, −*z*+1/2; (vi) −*x*+2, −*y*+1, −*z*+1; (vii) *x*+1, *y*, *z*.
